# Checkability and the Editorial Gate: Why Scientific Publishing Exempts Its Own Judgements From Scientific Standards

**DOI:** 10.1111/jep.70713

**Published:** 2026-10-28

**Authors:** Boris Nikolić

**Affiliations:** ^1^ Department of Philosophy Heinrich‐Heine‐Universität Düsseldorf Düsseldorf Germany

**Keywords:** accountability, desk rejection, editorial decision‐making, journal impact factor, peer review, publication ethics, research integrity

## Abstract

**Rationale, Aims and Objectives:**

The editorial gatekeeping of scientific publication is a chain of decisions: the editor's desk rejection, the recommendations of external reviewers, and the editor's adjudication of those recommendations, all shaped by journal metrics. The subjectivity of this process is well documented. This paper asks how discretion and accountability are distributed across the chain.

**Method:**

Conceptual and normative analysis organised around checkability, the standard science applies to its own claims: that a judgement's basis be recorded, its reliability measurable, and the judgement answerable beyond the say‐so of whoever made it. Each stage of the chain is assessed against this standard, using published evidence on editorial and reviewer agreement.

**Results:**

The editorial gate is exempt from checkability, and exempt by the greatest margin where it exercises the most discretion. The desk rejection, which disposes of most submissions at many journals, is a single editor's rapid decision on criteria acknowledged to be subjective, usually without a stated basis, external check or appeal. Reviewer recommendations whose agreement barely exceeds chance are aggregated into a single outcome. The adjudication of discordant reviews is again individual discretion, lightly checked. Existing codes govern editors' conduct, not the epistemic basis of their decisions.

**Conclusion:**

The wrong is not subjectivity, which is unavoidable, but an inversion: a system that demands checkability of the science it admits exempts from checkability the judging that admits it. The remedy is neither eliminating editorial judgement nor reviewing every submission, but holding the gate to the standard of checkability science demands of itself.

## 1 Introduction

Every published scientific paper has passed through a gate, and most submitted papers have been stopped at one. The gatekeeping of scientific publication, the process by which journals decide what to publish, is among the most consequential exercises of judgement in the research system: it determines what enters the record, shapes what is rewarded and therefore what is pursued, and, through the cumulative effect of countless individual decisions, channels the direction of entire fields. The process is conventionally described as peer review, but the term obscures more than it reveals, for the decision to publish is the product of a chain of distinct judgements made by different actors under different conditions: the editor's initial decision whether to review the manuscript at all, the recommendations of the external reviewers if it is reviewed, and the editor's final decision in light of those recommendations. This paper examines the chain as a whole through a single lens. Science holds its own claims to a standard that may be called checkability: the basis of a claim must be recorded, the reliability of the process that produced it must be measurable, and the claim must be answerable to scrutiny beyond the say‐so of whoever advanced it. Methods are reported so they can be examined, uncertainty is quantified, reliability is documented, and results are open to replication; a claim that cannot be checked is, by the discipline's own lights, not yet science. The argument of this paper is that the editorial gate which decides what becomes science is itself exempt from the standard of checkability, and that it is exempt by the widest margin exactly where it exercises the most discretion. The defining feature of the chain is therefore an inversion: the gatekeeping of science is held to a lower standard than the science it gates, and the discretion is greatest where the checkability is least.

That the process is subjective is not in dispute and is not the claim of this paper. The point has been made authoritatively and repeatedly. A systematic review of the evidence concluded that little empirical support exists for editorial peer review as a mechanism for ensuring the quality of research, and that the practice rests, in the reviewers' phrase, on faith in its effects rather than on facts [1]. To restate that conclusion would be to add nothing. The contribution here is structural rather than evidential: it concerns not whether the process is subjective, which is settled, but how subjectivity and accountability are distributed across the stages of the process, and what follows from the way they are distributed.

Two clarifications of scope and standpoint are owed at the outset. The first concerns scope. The argument is about the editorial gate of scholarly publishing in general, not about any particular journal. No journal's practice is examined here, and the evidence cited is drawn from the published literature on editorial decision‐making across several fields. The paper is addressed to a journal devoted to the evaluation of practice because the question it raises is an evaluative one: whether a practice that decides what counts as evidence meets the evidential standards it enforces. The second concerns standpoint. The author writes as a physician‐bioethicist in clinical practice who publishes in applied ethics and the philosophy of medicine, and who has served on editorial boards and as a peer reviewer of more than 300 manuscripts. He has therefore met the gate from both sides, as an author whose manuscripts have been accepted, returned for revision, and desk‐rejected, and as one who has assessed the submissions of others. That standpoint is declared so that readers can weigh it; the argument does not depend on it. Its claim is structural, concerning how discretion and accountability are distributed across the stages of the process, and it stands or falls on that distribution, not on the author's experience of any individual decision.

### 1.1 What Checkability Is

Checkability is the standard a discipline meets when the basis of a judgement can be inspected by someone other than the person who reached it. It has three components, each already familiar from the conduct of science itself. The first is a recorded basis: the grounds of a judgement are stated explicitly enough to be examined, rather than left implicit in the bare outcome. The second is measurable reliability: the process that produces the judgement is one whose consistency can be quantified and is known, so that the weight a judgement can bear is established rather than assumed. The third is the possibility of challenge: the judgement is answerable to review beyond the say‐so of whoever issued it, so that an error in its basis could in principle be identified and corrected. A judgement that meets all three is checkable; one that meets none is checkable in name only, however confidently it is delivered.

Why such decisions should be checkable at all follows from what they do. Decisions that allocate scientific attention, prestige, funding opportunities, and entry into the record are themselves epistemically consequential, and the more consequential a judgement is, the stronger the case that its basis should be inspectable, its reliability known, and its reasoning open to challenge. Editorial gatekeeping is among the most consequential forms of judgement in science; it follows that it requires not less checkability than the scientific claims it judges, but at least as much. The demand made here is therefore not that editorial judgement be eliminated or made mechanical, but that the most consequential judgement in the system not be the least answerable for itself.

These are not exotic requirements. They are the requirements science imposes on every empirical claim it admits: methods recorded so they can be scrutinised, reliability documented and inter‐rater agreement reported where judgement intervenes, and conclusions left open to challenge and revision. The argument that follows is that the editorial process which decides what becomes science satisfies none of the three with any consistency, and satisfies them least precisely where its decisions are most consequential. The remainder of the paper traces that failure stage by stage, beginning with the decision that disposes of the most submissions and is the least checkable of all.

### 1.2 Desk Rejection: Maximum Discretion, Minimum Checkability

The chain begins, for the majority of submissions, at the desk. At many journals the editor rejects a large fraction of manuscripts before they are sent for review at all, on the ground that they are unsuitable in subject, insufficiently novel, or unlikely to survive review; reported desk‐rejection rates range widely across disciplines but reach, at some journals, the substantial majority of all submissions [2, 3]. The practical case for the desk rejection is real and should be conceded at the outset: reviewer time is scarce, not every submission can be reviewed, and rapid triage spares reviewers unnecessary labour and authors a longer wait for an inevitable outcome. The objection is not to triage as such but to the conditions under which it is conducted. The desk rejection is the decision of a single editor, made quickly, often on the basis of the title, abstract, and a brief inspection; the criteria on which it turns, novelty, fit, priority, and likely interest, are acknowledged even by those who study editorial decision‐making to be soft criteria that are not objectively defined. When the consistency of such decisions has been examined directly, by having several editors of the same journal independently pre‐screen the same submissions, their agreement has been found to be modest, and the disagreement has been traced precisely to these subjective criteria [4]. The decision is, in the typical case, unaccompanied by any substantive statement of its basis, subject to no external check, and not open to appeal. The inconsistency is visible even in the form the decision takes. One author receives a considered paragraph identifying a specific deficiency; another, for a comparable manuscript at a comparable journal, receives a single templated sentence; a third receives no reason at all beyond a statement that the work will not be sent for review. That the same category of decision is discharged with such varying degrees of explanation, at the discretion of the individual editor and the pressures of the particular day, is itself evidence that no governing standard operates: where a standard governs a decision, it constrains not only the outcome but the account that must be given of it, and here neither the outcome nor the account is constrained. The gate that disposes of the most submissions is thus the one exercising the most unconstrained discretion, and it is also the one leaving the least trace by which the discretion could be reviewed.

### 1.3 Peer Review: Assessments of Unknown Reliability

The minority of manuscripts that pass the desk proceed to external review, on the premise that two or more qualified reviewers yield a more reliable assessment than one. That premise holds only if the reviewers' judgements are themselves reliable, and the evidence indicates they often are not: where reviewer agreement on the recommendation to reject has been measured with correction for chance, it has been found barely above chance, yet editors place substantial weight on those recommendations [5]. The point is not that reviewers disagree, which is unremarkable, but what the disagreement reveals: editorial decisions rest on inputs whose reliability is low and, more tellingly, unmeasured. Reviewers provide influential epistemic assessments that contribute to the editor's decision, but the reliability of those assessments is neither established nor disclosed, so the weight they can properly bear is unknown, a gap shown most directly by the rare experiment in which two independent committees judging the same submissions disagreed on the outcome for a substantial share of them [6, 7].

One might object that reviewers do not decide publication and so need not answer for their assessments. The objection misses the claim. Nothing here assumes that reviewers exercise decision‐making authority; the point is that editorial decisions depend on reviewer assessments whose reliability and weighting are ordinarily undisclosed, and a decision built on undisclosed and unmeasured inputs is to that extent uncheckable, whoever formally makes it. The issue is therefore not authority but checkability.

### 1.4 Editorial Adjudication: Discretion Without Checkability

The final decision belongs to the editor, who must resolve the reviewers' discordant recommendations into a decision. This adjudication is legitimate and necessary; reviewers advise and editors decide, and it could not be otherwise. The objection is not to editorial discretion, which evaluation cannot dispense with, but to discretion exercised without checkability: lightly constrained, seldom required to record its basis, and rarely answerable in terms a third party could examine. This discretion is also exercised within an incentive field, which the analysis should register but not lean on. Journals are judged largely by citation‐based metrics, so an editor has a structural interest in the metric editorial decisions affect, and the assessment of a manuscript's fit and likely interest is not plainly independent of its anticipated citations [8, 9, 10]. The claim is structural, not psychological, and its weight here is deliberately limited: whether or not the incentive bites in a given case, an uncheckable gate leaves the question unanswerable, since nothing in the record allows anyone to establish whether an exclusion reflected the work's merit or its unprofitability to the metric. The checkability argument does not rest on the incentive; the incentive is merely one further consideration that uncheckability keeps from view.

### 1.5 Governance of Conduct Versus Checkability of Judgement

Assembled, the chain exhibits a structure that a scientific community committed to its own evidential norms should find difficult to defend. At each stage the gatekeeping of science is conducted with more subjective discretion, and held to a lower standard of measured reliability and documented accountability, than that same community demands of the research passing through the gate. The system requires of its authors that they report their methods so that others may check them, that they quantify their uncertainty, that measurements be reproducible and inter‐rater agreement be reported where judgement is involved, and that claims be falsifiable and supported by stated evidence. It requires of its own gatekeeping almost none of this. The reliability of the desk decision is not measured; the agreement of reviewers is not reported to the authors and is, where measured, poor; the basis of the adjudication is not recorded in checkable form; and the whole operates under an incentive, the journal's metric, that is nowhere disclosed as a consideration. The wrong is not that judgement is exercised, for judgement is unavoidable and necessary in evaluation, nor even that the judgement is subjective, which all judgement is. It may be objected that the gate is not unaccountable at all: editors answer to editorial boards and publishers, and a substantial apparatus of guidance, the codes of bodies such as the Committee on Publication Ethics foremost among it, governs their conduct. The objection identifies something real but misses the distinction on which this paper turns. That apparatus governs the editor's *conduct*, prohibiting misconduct, requiring the disclosure and management of conflicts of interest, prescribing how allegations of fraud are handled; what it does not do is render the *epistemic basis* of the ordinary decision checkable. No code requires that the reason for a desk rejection be recorded in falsifiable terms, that the reliability of a journal's screening be measured, or that the weighing of discordant reviews be answerable to anyone beyond the editor who performs it. The gate is governed in the sense that its keepers are forbidden to be corrupt; it is uncheckable in the sense that matters here, that the exercise of its routine discretion need leave no checkable trace. The wrong is accordingly the inversion: that the most consequential and least checkable discretion in the scientific process is the discretion that polices the rest, and that a system demanding checkability of the work it judges exempts from checkability the judging itself.

### 1.6 Reforms: Making the Gate Checkable

The remedy that follows from this diagnosis is neither the elimination of editorial judgement nor the review of every submission. The first is impossible, since evaluation requires judgement, and the second is infeasible, since reviewer capacity is finite and triage is a genuine necessity. What the diagnosis calls for is the subjection of the gate to the standard the system already applies to its science: the standard of checkability. A gatekeeping decision is checkable to the degree that its basis is recorded, that the reliability of the process producing it is measured and known, and that the discretion it embodies is answerable to something beyond itself. Several measures follow without any need to abolish editorial discretion. The mundane and objective components of the desk decision, completeness, adherence to scope, the absence of plagiarism and of ethical defect, can be separated from the subjective judgement of novelty and interest, standardised, and in many cases automated, so that the genuinely discretionary part of the decision is isolated and named as such [11]. The basis of a desk rejection can be stated to the author in terms specific enough to be assessed, rather than in the unfalsifiable language of fit and priority. The reliability of a journal's editorial process, the agreement of its reviewers and the consistency of its pre‐screening, can be measured and, in principle, reported, as a property of the journal that authors and readers are entitled to know. And the incentive that the metric creates can be disclosed and, where possible, insulated from the assessment of individual manuscripts. None of these makes the gate objective, which it cannot be; each makes it accountable, which it can.

## 2 Limitations

Several limitations qualify this argument. First, it is conceptual rather than empirical: it draws on existing studies of reviewer agreement, editorial inconsistency, and the contingency of acceptance, but it does not itself measure the checkability of any gatekeeping process, and the central claim, that the gate is held to a lower standard of checkability than the science it admits, is advanced as a structural characterisation rather than a quantified finding. Second, the evidence on which the reliability argument rests, though consistent in direction, is limited in volume and drawn substantially from particular journals and a single conference experiment, and the generalisability of those findings across fields and venues is not established. Third, the claim that impact‐factor incentives distort the published record is, as the text states, a structural conjecture about the direction of an incentive and not a demonstration of its magnitude in any decision; the counterfactual it depends upon, the fate of work that the gate excluded, is by its nature difficult to observe. Fourth, the constructive proposal, that gatekeeping decisions be made checkable, is offered as a principle rather than a validated intervention, and whether the specific measures suggested would improve the reliability or fairness of editorial decisions, and at what cost in editorial labour and timeliness, is an empirical question this paper frames but does not answer. These limitations bound the argument; they do not, in the author's view, undermine its central observation, that a system demanding checkability of its science exempts from checkability the gate that admits it.

The natural continuation of this argument is empirical, and the paper is intended as the opening of that inquiry rather than its conclusion. Three lines of work would test and refine it. The first would measure checkability directly, by examining for a sample of journals whether desk‐rejection decisions record a basis, whether the consistency of screening is measured, and whether reviewer agreement is known to the editors who rely on it. The second would seek the perspectives of those who work within the gate, through surveys and interviews with editors, members of editorial boards, reviewers, authors, and publishers; the conditions under which editors work, including high and rising submission volumes and editorial labour that is often unpaid, bear directly on which reforms are feasible, and a structural argument cannot settle that question from the outside. The third would examine the growing use of automated and AI‐assisted tools in screening and triage, which cuts in both directions: such tools can record the basis of a decision more consistently than a hurried editor, but they can also introduce criteria that are neither disclosed nor examined, and so either extend or erode the checkability of the gate. Each would convert a structural characterisation into a measured one; each is framed, though not undertaken, here.

## 3 Conclusions

The argument of this paper has been that the editorial gatekeeping of science fails not because it is subjective, which is unavoidable, but because its subjectivity is distributed in inverse proportion to its accountability, so that the most discretionary decisions are the least checkable, and the whole is held to a standard of rigour far below the one it enforces upon the research it judges. The desk rejection, the reviewer aggregation, and the editorial adjudication are each defensible as functions and each unchecked as exercises of discretion, and the metric that shapes them is the same one the research‐evaluation system has been faulted for misusing elsewhere. A scientific community that demanded of its own gatekeeping what it demands of its science, that the basis of judgement be recorded, that reliability be measured, and that discretion be answerable, would not thereby make its gate objective, but it would make it a part of the scientific enterprise rather than an exception to it. The gate would be governed, and a process that determines what counts as scientific knowledge would at last be held to the standards of the knowledge it admits.

## Author Contributions

Boris Nikolic is the sole author; he conceived, drafted, and revised the manuscript and approved the final version.

## Funding

The author has nothing to report.

## Ethics Statement

The author has nothing to report.

## Conflicts of Interest

The author declares no conflicts of interest.

## Declaration of Generative AI Use

During the preparation of this work the author used Claude (Anthropic) to assist language editing, formatting and manuscript organization. The tool does not meet the journal's authorship criteria and is not listed as an author.

## Data Availability

The author has nothing to report.
